# Omics Meeting Onics: Towards the Next Generation of Spectroscopic-Based Technologies in Personalized Medicine

**DOI:** 10.3390/jpm9030039

**Published:** 2019-08-01

**Authors:** Weng Kung Peng, Daniele Paesani

**Affiliations:** Precision Medicine–Engineering Group, Department of Nanoelectronics Engineering, International Iberian Nanotechnology Laboratory, 4715-330 Braga, Portugal

**Keywords:** personalized medicine, spectroscopic-based technologies, decentralization, technology democratization

## Abstract

This article aims to discuss the recent development of integrated point-of-care spectroscopic-based technologies that are paving the way for the next generation of diagnostic monitoring technologies in personalized medicine. Focusing on the nuclear magnetic resonance (NMR) technologies as the leading example, we discuss the emergence of -onics technologies (e.g., photonics and electronics) and how their coexistence with -omics technologies (e.g., genomics, proteomics, and metabolomics) can potentially change the future technological landscape of personalized medicine. The idea of an open-source (e.g., hardware and software) movement is discussed, and we argue that technology democratization will not only promote the dissemination of knowledge and inspire new applications, but it will also increase the speed of field implementation.

Point-of-care (PoC) personalized medicine is one of the cornerstones of the next generation of precision health and medicine. When the first human genome project was initiated in the early 1990s, extensive effort was placed on mapping the role of genes in the onset of diseases. Until recently, personalized medicine has always been associated with genomic medicine because of the ´hypes´ of providing a molecular basis of health and diseases, and in this way allowing disease stratification and preventive medicine in a personalized manner. Genetic contributions to different diseases, however, were found to be varied and often had very little impact, with non-genetic factors such as macroenvironment (e.g., environmental hazards) and microenvironment (e.g., microbiome) having much greater attributable risks.

As the human genome project drew to an end, new waves of technological advances in the field of genomics (e.g., gene editing [1,2], 3D genomics [3,4], functional genomics [5]) and epigenomics [6] (e.g., spectroscopic-based (molecular phenotyping) technologies [7,8]) began to emerge. Focusing on the nuclear magnetic resonance (NMR) technologies as the leading example, we discuss the emergence of -onics technologies (e.g., photonics and electronics) and how their coexistence with -omics technologies (e.g., genomics, proteomics, and metabolomics) can potentially change the technological landscape of personalized medicine.

Functional genomics focuses on dynamic aspects such as gene transcription and protein–protein interactions, as opposed to the static aspects of genomic information. Molecular phenotyping (or epigenetic in a narrower sense), on the other hand, focuses on the interaction from a single gene (or specific protein) to a much larger biological scale (e.g., liquid biopsies) with respect to its macro/micro environmental effect, in which studied can be made using the analytical tools (spectroscopic-based technologies). The result of molecular spectroscopy, especially in the higher dimensions (2D and beyond), forms unique biomarkers (´molecular fingerprint´) (time)-specific to the biological footprint at the molecular level (Figure 1). This unique ´molecular fingerprint´ is often acquired independent of the -omics database, which, interestingly, create new opportunities for understanding new or formerly nonexistent biological pathways.

Among the spectroscopic-based technologies, impedance spectroscopy has emerged as one of the most promising candidates for the next generation of low-cost and integrated PoC diagnostic monitoring technologies in personalized medicine. Interaction between the electromagnetic fields and matter produces responses as a result of the absorption, reflectance, or transmission of physical properties. In particular, dielectric spectroscopy (e.g., NMR spectroscopy) and THz spectroscopy provide powerful techniques that exploit the nonionizing portion and nondestructive nature of the electromagnetic spectrum.

The concept of decentralization lies in the heart of personalized medicine. In recent years, significant advances in the semiconductor industry (e.g., complementary metal-oxide-semiconductor (CMOS), field programmable gate arrays (FPGA)) enabled the emergence of ultra-low-cost NMR technologies (e.g., spectroscopy, relaxometry, and imaging) in research environments along with their respective open-source codes. Semiconductor technology enables low-cost and large-scale integration of transistor arrays and physical sensing materials on a single chip, combining digital signal transducing/processing with peripheral analogue circuitry into a single board, thus leading to the first wave of low-cost, portable NMR-based in vitro diagnostics in point-of-care settings [9,10]. These include immuno-magnetic particle labeling-based tumor detection (e.g., breast cancer [11], epithelial [12,13], melanoma [14]), and bacterial detection (e.g., tuberculosis [15], *Escherichia coli* [16])); label-free detection, such as blood oxygenation [17] and oxidation [18] levels, malaria screening [19,20,21]; and rapid phenotyping of diabetes mellitus [7,22,23].

Unlike the more expensive ex vivo clinical imaging modalities, the new paradigm of lowered engineering barriers (e.g., lower cost, lower power use, higher portability) while tapping on the noninvasive properties of magnetic resonance has opened new avenues for in vitro diagnosis of liquid biopsies. Direct, nondestructive cellular characterization has significant implications, as each individual cell can be viewed as a complex mechanistic machine with unique (set of) characteristics (phenotype), thus providing a snapshot of information that serves as a direct proxy to the health status of an individual (genotype) with respect to their macro/microenvironment (exposome). One of the paradigms in personalized medicine is the promise of providing disease management (e.g., diagnosis, prognosis, and predictive treatment/recurrence) in a uniquely personalized manner, as opposed to the traditional ´one size fits all´ model.

The nondestructive capability of NMR allows the design of functional assays, in which unique and specific responses can be obtained in qualitative and quantitative manners. This will be useful for disease prognosis (or monitoring) and relevant to modern human´s chronic diseases (e.g., cancer, diabetes mellitus), where other complication(s) may develop as the consequence of a primary disease (patho-phenotype). Another option is to move towards high-throughput single cell characterization, which has a higher spatiotemporal resolution (as compared to bulk measurement). This will be an element of primary importance in driving evidence-based medical decisions, as data science and/or artificial intelligence pattern recognition will enable complete mapping of single cell analyses, offering arguably the best medical basis for decision making [24].

More recently, a second wave of ultra-low-cost NMR technologies has seen the emergence of software based radio (SDR)-based spectrometers, which exploit the open-source environment (e.g., GNU radio). Such a movement has potential to shift the research community and society at large towards technology democratization, thus departing from the (over)-dependency on expensive commercial spectrometers. Technology democratization will not only promote the dissemination of knowledge and inspire new applications, but it will also increase the speed of field implementation.

SDR ranges from hobbyist levels that are extremely low cost (e.g., rtl-SDR, Hack RF), to medium cost (e.g., LimeSDR, kiwi-SDR), and to professional instrumentation (e.g., Ettus Research). A typical SDR-based spectrometer comprises a basic front-end controller/tuning and a digital section (e.g., transceivers, pulse programmer) separated from the analogue section (e.g., rf-probe, duplexer, power amplifier, low-noise amplifier) (Figure 2). SDR enables the replacement of traditional hardware components of an RF system with software-based signal processing, thus allowing mixing, filtering, detection, modulation, and any other signal generation/detection steps to be carried out without the need for specialized analog hardware. This technology potentially enables the implementation of highly versatile, wide-band and wide-spectrum RF systems, which disregard the strict limitations of hardware in tuning and detection, yielding a seamless integration towards any subsequent signal analysis step with continuously reprogrammable transmitter and receiver solutions.

The large bandwidth (from low kHz to GHz) makes it attractive to the amateur community, as it allows low- to high-field NMR spectroscopy, ESR spectroscopy, and electron-nuclei double resonance (ENDOR) analyses to be carried out at ease. NMR spectroscopy is known to detect protein and metabolites, while ESR spectroscopy is able to directly detect free radicals, which play a major role in many chronic diseases and cell aging [25,26]. ENDOR will allow communication between the electron and nuclei to be established.

Most importantly, a vast set of dedicated open source software is available, which ranges from general purpose RF scanners (SDR-Sharp, Cubic SDR) to more specialized suites for aerospace, telecommunications, and analytical (imaging) technologies (gR-MRI). These packages, paired with high-end SDR hardware systems (e.g., Ettus Research SDR), can provide a significant improvement for spectrometry research at much lower costs relying on potentially more powerful/versatile instruments. One advantage of open-source technologies is their high possibility of reconfiguration and adaptability to the need of each experiment, which may not be possible in ´black box´ environments in commercial systems.

One of the earliest SDR-based NMR spectrometers can be traced back to 2008 when Takeda exploited FPGA to run the entire digital section of the spectrometer [27]. Three years later, Tang et al. demonstrated that 1D and 2D relaxometry can be performed using a single-board NMR spectrometer based on a software-defined radio architecture [28]. The SDR-based spectrometer was realized by combining FPGA and a digital signal processor chip with peripheral radio frequency front-ends. Asfour et al. implemented the idea of down conversion with a direct digital synthesizer and observed free induction decays [29]. Their SDR includes direct analog-to-digital conversion and a digital down conversion (e.g., digital quadrature demodulation, decimation filtering, processing gain). Recently, Hasselweander et al. used two sets of SDRs, known as universal software radio peripheral 1 (USRP1), and demonstrated imaging capabilities [30]. One SDR acted as a transceiver while the other was used for gradient field generation. The USRP family is designed by the Ettus Research of National Instruments for accessibility, and many of its related products are open-source hardware. The authors have shared the open source software (gr-MRI) online. Recently, Michal has shown that parallelization of multichannels was possible with low-cost SDR transceivers. The digital pulse programmer is powered by an ultra-low-cost microcontroller (e.g., Arduino). Multichannel parallelization of heteronuclear magnetic resonance sensors was also demonstrated by Huber et al. [31].

Early history of software-defined radio started from wireless radio communications, which first saw the implementation of reconfigurable short-wave receivers in the early 80s. What was once an expensive semiconductor chip with a technology readiness level (TRL) of 1–2 in its early infancy, the semiconductor industry has boosted telecommunication technology to much higher TRLs, has made it incredibly low cost to consumers, and, consequently, has created an affordable platform for hobbyists today. Interestingly, the reconfigurability options of this technology allow creativity to stretch beyond our imagination, and unexpected, new inventions continue to be developed beyond its original field (such as the crossover of radio telecommunication field) to personalized medicine.

Technology democratization will bring about a new wave of social liberation because of the growing segmentation of the ultra-low-cost market, which has formerly been nonexistent. From Wikipedia to Arduino, open-source platforms have inspired and promoted the idea of sharing and is perhaps one of the greatest gifts to the next generation. With ubiquitous access to internet (e.g., open-access articles) and low-cost fabrication of hardware (e.g., 3D printing), more engineering barriers that we once had will be taken down. In this way, the process of technology democratization will help to further ´flatten´ society [32] or/and may become killer strategies in view of tight environmental funding in many years to come.

In conclusion, we welcome a new era where the melting pot of omics and onics technologies meet as we move forward enthusiastically towards the next generation of spectroscopic-based technologies in personalized medicine.

## Figures and Tables

**Figure 1:**
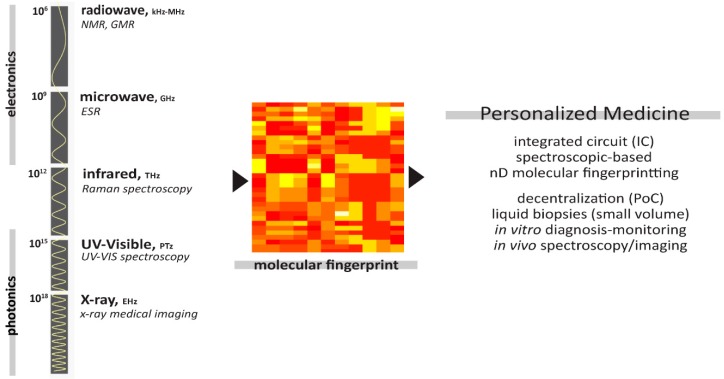
Electromagnetic spectrum with respect to the impedance spectroscopy used (e.g., nuclear magnetic resonance (NMR), electron spin resonance (ESR), and THz spectroscopy) to develop the next generation of integrated PoC spectroscopic-based technologies in personalized medicine.

**Figure 2:**
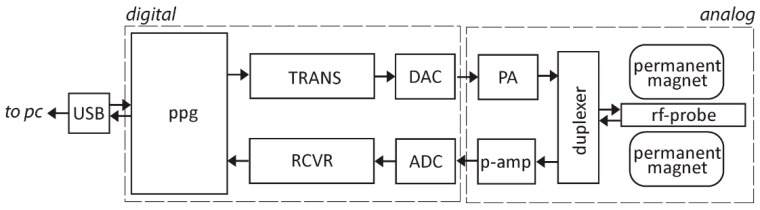
A simplified block diagram of the architecture of a software based radio (SDR)-based NMR spectrometer showing the separation of digital and analogue sections. The glossary is as follows: pulse programmer (PPG), receiver (RCVR), transmitter (TRANS), analog-digital converter (ADC), digital-analog converter (DAC), power amplifier (PA), preamplifier (p-amp), radio frequency (rf), and universal serial bus (USB).
